# The Right to Food for Children With Cerebral Palsy in South Africa: Examining Nutritional Access and Structural Barriers

**DOI:** 10.1111/cch.70323

**Published:** 2026-08-23

**Authors:** Tiya Vala, Radia Ameen, Skye Nandi Adams

**Affiliations:** ^1^ Department of Speech Pathology and Audiology University of Witwatersrand Johannesburg South Africa

**Keywords:** cerebral palsy, feeding practices, nutritional needs, South Africa

## Abstract

**Background:**

Children with cerebral palsy (CP) are at increased risk of malnutrition due to feeding difficulties, higher nutritional requirements and the additional caregiving demands associated with disability. Households caring for children with CP may therefore experience greater vulnerability to food insecurity, particularly in low‐resource contexts. This study explored how children with CP experience feeding and nutrition within the South African food environment.

**Methods:**

An explanatory mixed‐methods pilot study was conducted with primary caregivers of children aged 2–8 years with CP in South Africa. Caregivers completed a descriptive survey assessing household food access, feeding practices and child functional severity using the Gross Motor Function Classification System (GMFCS) and Eating and Drinking Ability Classification System (EDACS). Semi‐structured interviews were subsequently conducted to explore feeding experiences. Data were interpreted using the food security framework.

**Results:**

Although food was generally present within households, caregivers reported limited access to foods that met the nutritional and feeding needs of children with CP. Feeding required extensive caregiver adaptations, including texture modification and prolonged mealtimes. Economic constraints, reliance on social grants, transport barriers and unstable income further affected household food access and stability. Children with greater feeding impairment experienced more pronounced challenges in achieving safe and nutritionally adequate diets.

**Conclusions:**

Children with CP experience intersecting vulnerabilities across the dimensions of food availability, access, utilization and stability. Disability‐responsive nutrition policies, strengthened caregiver support and integrated health and social protection strategies are needed to support equitable access to adequate food for children with CP.

## Summary

- Feeding challenges for children with cerebral palsy extend beyond clinical swallowing impairments and are shaped by structural conditions and caregiver knowledge that influence the availability, affordability and preparation of appropriate foods within households.
- Children with greater feeding impairment experience increased difficulty achieving safe and nutritionally adequate diets, with food affordability emerging as a key determinant of dietary quality and food choices.
- A food sovereignty approach, alongside disability‐inclusive nutrition policies and integrated health and social protection systems, is needed to improve food security and nutritional outcomes for children with cerebral palsy.

## 1 Introduction

Cerebral palsy (CP) is a leading cause of childhood physical disability and is consistently associated with an elevated risk of malnutrition (Aggarwal et al. 2015; Vova 2022). Children with CP frequently experience feeding and swallowing difficulties, gastrointestinal complications and motor impairments that limit their ability to chew, swallow and safely consume food (Da Silva et al. 2022; Calderone et al. 2025; Dalpatadu et al. 2025). Oropharyngeal dysphagia, reported in a substantial proportion of this population, contributes to prolonged mealtimes, reduced dietary intake and increased risk of aspiration, while conditions such as gastro‐oesophageal reflux and constipation further compromise nutritional status. These clinical and functional challenges mean that adequate nutrition for children with CP depends not only on the presence of food but also on its texture, preparation, feeding support, posture, mouth control and safety during consumption.

### 1.1 The Fundamental Right to Be Free From Hunger

In response to persistent global food insecurity, the United Nations introduced Sustainable Development Goal (SDG) 2: Zero Hunger, which aims to eliminate hunger, achieve food security, improve nutrition and promote sustainable agriculture by 2030 (Arora and Mishra 2022). Despite global progress, food insecurity and malnutrition remain ongoing public health concerns across many settings (Viana et al. 2021). Although SDG 2 is universal in scope, emerging scholarship suggests that disability considerations are not consistently foregrounded within food security initiatives, raising questions about the extent to which children with disabilities, including those with CP, are represented within policy and program responses.

Within low‐and middle‐income contexts (LMICs) such as South Africa, these feeding complexities intersect with broader socioeconomic constraints. Households caring for children with CP often incur additional costs related to specialized feeding equipment, modified diets, nutritional supplements, healthcare utilization and transport (Snik et al. 2019; Kibrom et al. 2024; Melak et al. 2025; Naidoo et al. 2024). As a result, children with CP may experience functional hunger, defined as the inability to effectively ingest or utilize adequate nutrition despite food being present within the household (Kuperminc et al. 2013; Sleigh 2005). Hunger in this context reflects barriers to accessing food that is nutritionally dense, appropriately textured and safely consumable, highlighting the multidimensional nature of food insecurity for this population.

Food security provides a useful framework for understanding these challenges. Conceptualized across four interrelated pillars: availability, accessibility, utilization and stability. Food security extends beyond food supply to encompass the ability to obtain, prepare, consume and consistently access adequate nutrition (Ingram 2020; Xie et al. 2021). For children with CP, the utilization dimension is particularly salient, as nutritional adequacy depends on caregiver capacity, caregiver knowledge regarding safe feeding management affected by specific dysphagia‐related disorders, feeding skills, time, adaptive equipment and clinical support to ensure safe and effective feeding (Mbhenyane and Tambe 2024; Nyman et al. 2025; Parkes et al. 2010). Disruptions across these pillars therefore shape not only household food security but also children's nutritional wellbeing and health. These concerns must be understood within a human rights framework. The right to food, protected within national and international legal instruments, requires that food be available, accessible, acceptable and adequate for all individuals without discrimination. For children with CP, realization of this right is often constrained by feeding impairments, resource limitations and health system gaps that restrict meaningful access to safe and nutritionally appropriate food (Makhoul et al. 2026). Despite growing recognition of disability‐related nutritional vulnerability, there remains limited empirical evidence describing how caregivers of children with CP in South Africa experience food insecurity and negotiate feeding within constrained environments.

### 1.2 A Disability‐Inclusive and Rights‐Based Perspective on Food Security

Limited research has examined the forms of support required by caregivers to meet the feeding and nutritional needs of children with CP within contexts of food insecurity (Jahan et al. 2022; Ostojic et al. 2025). This gap is particularly notable in Sub‐Saharan Africa, where there is a paucity of evidence describing caregiver experiences related to food procurement, dietary modification and feeding practices for children with CP (Melak et al. 2025). Within South Africa, social protection mechanisms such as the disability grant are intended to support households caring for children with disabilities; however, little empirical work has explored how such support intersects with the specialized feeding and nutritional needs associated with CP (Adams et al. 2024; Mbhenyane and Tambe 2024). Consequently, the extent to which existing food security strategies and social supports adequately respond to disability‐related nutritional requirements remains unclear. These knowledge gaps highlight the need for research that examines food security and feeding through a disability‐inclusive and rights‐oriented lens. Understanding caregiver experiences is essential for informing policies and services that aim to support equitable access to adequate and appropriate nutrition for children with CP. Without contextually grounded evidence, it is difficult to determine how global commitments such as SDG 2 translate into meaningful nutritional inclusion for this population.

Ensuring freedom from hunger for children with CP therefore requires a rights‐based, disability‐inclusive approach that recognizes their complex feeding needs, addresses economic and physical barriers to food access and strengthens caregiver support systems. Without such targeted strategies, the global commitment to zero hunger will continue to overlook one of the most nutritionally vulnerable child populations. Therefore, this study explores caregiver experiences of feeding children with CP in South Africa through a food security lens, examining how intersecting challenges related to availability, access, utilization and stability shape the realization of the right to food for this population.

## 2 Methods

### 2.1 Study Design

An explanatory mixed‐methods design was used, consisting of a quantitative descriptive survey followed by qualitative semi‐structured interviews. This design enabled the identification of patterns in feeding challenges (Phase 1) and provided in‐depth contextual explanations of these patterns through caregivers' narratives (Phase 2). The quantitative phase included standardized tools assessing socio‐demographic characteristics, functional severity, eating and drinking ability and household food access. The qualitative phase, guided by phenomenological principles, explored caregivers' lived experiences, decision‐making processes and interpretations of the feeding challenges identified in the survey. This sequential approach was appropriate for a pilot study and ensured methodological rigour by linking measurable trends to subjective, context‐dependent experiences. This study was approved by the University of the Witwatersrand Human Research Ethics Committee (HREC) (Non‐Medical) (Approval number: STA_2025_39). Informed consent was provided by all participants prior to completion of the online survey and again before the interview.

### 2.2 Participants and Recruitment

Non‐probability convenience sampling was utilized. Recruitment took place at two physical disability‐focused centres and through various social media platforms (WhatsApp, Instagram and Facebook). Using social media expanded accessibility to caregivers who faced time, transport or caregiving constraints, aligning with research indicating that digital platforms are increasingly valuable for reaching dispersed or under‐resourced populations. This online site played a critical role in the recruitment process and supported inclusivity by enabling participation beyond physical care centres. By incorporating both disability‐focused centres and social media settings, the study captured caregiving experiences situated within varied socio‐economic conditions and service contexts.

Eligible participants were primary caregivers of children aged 2–8 years with a confirmed diagnosis of CP. Caregivers were required to be directly responsible for the child's daily feeding and nutritional decision‐making. Caregivers also needed to be proficient in English to participate in interviews. Children across all levels of the Gross Motor Function Classification System (GMFCS) and Eating and Drinking Ability Classification System (EDACS) were included.

### 2.3 Survey Development

A study‐specific survey (Supporting Information) was developed by the three authors who are all speech‐language pathologists working with children with CP and associated feeding difficulties in South Africa. The third author (SA) is a lecturer at the University of the Witwatersrand and her research focuses on paediatric feeding difficulties and family‐centred approaches to intervention. Research Electronic Data Capture (REDCap) was used for screening, electronic consent and survey administration (Patridge and Bardyn 2018). Caregivers were asked to consult their child, as appropriate. The survey included five data collection tools. Following the completion of the survey, all participants were interviewed online.

#### 2.3.1 Demographic Questionnaire

The demographic questionnaire captured caregiver demographics, socio‐economic conditions and child‐related information, providing essential context for interpreting feeding practices and barriers within the South African food environment.

#### 2.3.2 GMFCS

The GMFCS is a functional classification system composed of five levels, used to assess the motor skills of children with CP. The levels are I: walks without limitations, II: walks with limitations, III: walks with the aid of a manual mobility device, IV: has limited mobility and can use a motorized chair and V: needs to be transported in a manual wheelchair (Ho et al. 2017). Based on this system, scores one and two are defined as mild, three is moderate, and four and five are classified as severe CP (Rosenbaum et al. 2008).

#### 2.3.3 EDACS

The EDACS was also used as an instrument for screening to categorize each child's eating and drinking ability. Scores ranged from minimal difficulty to severe impairment with aspiration risk. This facilitated comparisons between caregiver experiences across levels of feeding severity. The EDACS was studied and proven to be valid and reliable (Tschirren et al. 2018).

#### 2.3.4 Food Inventory Questionnaire

The Food Inventory Questionnaire was used to assess household food availability and children's dietary intake, with analysis focused on whether specific foods were eaten by the child or not. It was adapted to the South African context using locally relevant foods (e.g., pap, amasi, samp, vetkoek), informed by *Eating and Drinking in Southern Africa* (Du Rand and Fisher 2020), enabling a culturally and environmentally valid assessment of feeding within household food constraints.

#### 2.3.5 Household Food Insecurity Access Scale (HFIAS) for Measurement of Food Access Questionnaire

This tool was used to better understand food security in each of the families' households and was adapted from approaches used in the United States of America (Coates et al. 2007). HFIAS was developed as a guide to understanding the multidimensional concept of food insecurity (Coates et al. 2007). This questionnaire guided interviews related to food security under different domains. This questionnaire was used in Ethiopia and developing countries (with different cultural contexts) in recent years to gather insight into food insecurity and household perceptions of this (Coates et al. 2007). The prevalence of household food insecurity can be determined by the use of the HFIAS. Gebreyesus et al. (2015) found evidence that the HFIAS tool has good internal consistency is a valid tool to measure household food insecurity. It is recommended that adaptations be made to the wording of the tool before its use (Gebreyesus et al. 2015). It was found that the HFIAS was reliable and valid when translated into Arabic and used in rural Lebanon (Naja et al. 2015).

#### 2.3.6 Semi‐Structured Interviews

Semi‐structured interviews were conducted with caregivers following the completion of the online survey. The interview questions focused on caregivers' lived experiences, daily routines, strategies, safety concerns and socio‐cultural influences on feeding. Interviews were conducted online via Microsoft Teams, with 1GB mobile data provided where necessary. Each interview lasted between 45 and 55 min. All participants signed an additional consent form prior to participation.

### 2.4 Data Analysis and Integration

To ensure accuracy, two researchers independently checked and entered the data (TV, RA). The characteristics of the recruited children and caregivers were expressed as frequencies and percentages. The survey data were analysed descriptively. For all participants, GMFCS, EDACS and HFIAS scores were calculated and reported. Interview data were analysed using thematic analysis. Thematic analysis has six steps: (1) familiarization of the data, (2) coding the data, (3) generating themes, (4) review and development of themes, (5) defining and refining themes and (6) writing the report. Two researchers (TV, RA) independently coded all responses. Discrepancies were resolved by consensus with adjudication by a third independent coder (SA). Themes were discussed with all authors, and key themes were triangulated with the survey data. Themes were derived both inductively and through predetermined areas of discussion, based on known feeding challenges from the literature. Quantitative and qualitative datasets were integrated during analysis. Survey findings provided descriptive patterns of feeding challenges and food access, while qualitative data contextualized and explained these patterns. This integrated analysis improved the depth, validity and interpretive richness of findings for a pilot investigation.

## 3 Results

### 3.1 Participants

Nine children with cerebral palsy were included in the study (Table 1). Their primary caregivers participated as respondents, with five children recruited through disability centres and four through social media.

**TABLE 1:** Child characteristics.

	Age (years)	Gender	GMFCS level	EDACS rating
Child 1	6	Male	3	2
Child 2	4	Female	4	2
Child 3	3	Female	4	2
Child 4	7	Male	4	3
Child 5	5	Male	^a^	5
Child 6	2	Female	5	3
Child 7	5	Female	5	5
Child 8	3	Female	5	3
Child 9	8	Male	5	4

^a^ Indicates that participants did not include this information.

The children represented in this study ranged in age from 2 to 8 years with the majority of children being (*n* = 5, 55.6%). Children's functional classifications reflected moderate to severe motor and feeding impairments. GMFCS levels ranged from level 3 to level 5, with level 5 being the most common. EDACS levels ranged from level 2 to level 5, with level 2 most frequently reported. Level 2 indicates that children can eat and drink orally with some assistance, whereas level 5 reflects the need for alternative, non‐oral feeding methods, such as a percutaneous endoscopic gastrostomy (PEG).

Caregiver characteristics are presented in Table 2. Caregivers in the study were all female (*n* = 9, 100%) with an average age of 38.1 years ± 9.69 (range 28–57 years). Most of the caregivers were mothers (*n* = 8, 88.9%) with one being a grandmother, and five (55.6%) participants reported being employed full time.

**TABLE 2:** Caregiver characteristics.

Caregivers	Age (years)	Relationship to child	Employment status	Monthly household income (USD)^a^	Number members in household including child and caregiver	Government grant^b^
Participant 1	39	Mother	Employed full‐time	125–312.50	3	Government grant—not specified
Participant 2	29	Mother	Employed full‐time	625–1250	5	No
Participant 3	49	Mother	Employed full‐time	More than 1250	4	Government grant—not specified
Participant 4	39	Mother	Employed part‐time	125–312.50	4	Government grant—Child grant and care dependency grant
Participant 5	57	Grandmother	Unemployed	125–312.50	6	No
Participant 6	28	Mother	Employed full‐time	312.50–625	5	Government grant—Child grant and care dependency grant
Participant 7	37	Mother	Unemployed and seeking employment	Less than 125	4	No
Participant 8	29	Mother	Unemployed	Less than 125	3	No
Participant 9	36	Mother	Employed full‐time	More than 1250	3	No

^a^ Exchange rates were converted from South African Rand (ZAR) to United States Dollars (USD) using an average rate from 2025 rate of 1 ZAR = $0.056 USD (equivalent to 1 USD ≈R17.88).

^b^ Caregivers were asked if they received any government grants. The Child Support Grant is managed by the South African Social Security Agency (SASSA) and is a means‐tested government grant provided to caregivers of children to assist with basic needs. Caregivers receive $31.36 (R560.72/month) (South African Government 2014). The Care Dependency Grant is provided to caregivers of children with severe disabilities requiring permanent care or support. Caregivers receive a monthly payment of $129.92 (R2 322.97/month). The care dependency grant covers disabled children from birth until they turn 18.

Five children (55.6%) resided in households reporting an estimated monthly income of $312.50 or less (≤ R5000), and four households reported receiving some form of government grant support. Households with reported incomes between $125 and $625 per month (R2000–R10,000) were receiving government grants, whereas the two households reporting the lowest income bracket (less than $125 per month [< R2000]) were not receiving any grant support. Household size ranged from 3 to 6 members, with a mean of 4 additional members residing in each household. During the study period, South Africa's national minimum wage was approximately $1.72 per hour (R27.58/h), equivalent to roughly $275 per month (R4400/month) for full‐time employment (40 h per week) (National Minimum Wage Commission 2024). Several households therefore reported incomes at or below minimum wage levels. Food security status was assessed using the HFIAS, and results are presented in Table 3.

**TABLE 3:** HFIAS Scores for each participant and their household.

Participant number	HFIAS score	Category of food insecurity
1	1	Food secure
2	0	Food secure
3	3	Mildly insecure
4	7	Severely insecure
5	0	Food secure
6	0	Food secure
7	4	Moderately insecure
8	14	Severely insecure
9	0	Food secure

*Note:* HFIAS scores range from 0 to 27, with higher scores indicating greater household food insecurity. Classification into food secure, mildly food insecure, moderately food insecure and severely food insecure categories was based on standard HFIAS scoring criteria, which consider both frequency and severity of food access experiences over the preceding 4 weeks.

Five households (55.6%) indicated that they were food‐secure households. One household was deemed mildly food insecure, and another one was deemed moderately food insecure. The two remaining households had scores that placed them in the category of severely food insecure.

### 3.2 Food Availability

All 9 households (100%) relied on purchased food as their primary food source with no caregivers reporting consistent access to home‐grown produce. Physical access to food outlets was generally reported as adequate, with most caregivers living near formal retailers. At the retail level, caregivers reported that a wide variety of foods was generally available through formal supermarkets, butchers and fresh produce markets. Most participants described accessing food from supermarkets such as Pick n Pay, Shoprite and Woolworths, and did not report household‐level shortages related to storage or food preservation. However, retail availability was seen as a primary concern along with financial and structural constraints. These limited caregivers' ability to utilize available food resources, ‘It's not just the money. Sometimes I cannot find the food that I need for my child to eat’ (P3). Although a variety of food was available to purchase, seven caregivers (77.8%) reported that available foods were often not appropriate for their child's feeding needs due to texture or preparation requirements. One caregiver explained, ‘He doesn't really eat a lot of things … he doesn't eat meat because sometimes when you blend the meat, he won't eat it’ (P8). This highlights how functional feeding limitations restricted the practical usability of otherwise available foods. These accounts suggest that while food may be physically present within the broader food environment, economic access and retail supply variability shaped household‐level availability.

### 3.3 Food Access

Six caregivers (66.7%) reported limited availability of fresh and nutritionally diverse foods within their local environments. The food inventory questionnaire was used to determine which foods were eaten by the child to illustrate the range of foods and types of foods consumed. Table 4 shows the different foods and food groups eaten and those that are eaten the most and least. Although children ate a variety of foods, affordability ultimately determined household food choices. Caregivers consistently emphasized that food purchasing decisions were shaped not by nutritional preference or suitability, but by cost. As one caregiver stated, ‘You buy what is there and what you can afford’ (P3), while another reflected, ‘Most of the food around here is bread and pap’ (P5). These accounts highlight that although food variety exists within the retail environment, economic constraints narrow practical options, particularly limiting access to nutritionally important foods.

**TABLE 4:** Foods eaten by children with CP (*N* = 9).

Food group	Food item (%)
Meats, fish and proteins	Beef (100.0), Chicken (100.0), Eggs (100.0), Beef burgers (77.8), Chicken nuggets (77.8), Bacon (77.8), Sausages (77.8), Boerewors (77.8), Fish fried in batter/breadcrumbs (77.8), Tinned tuna (77.8), Lamb (66.7), Ham (66.7), Pork (55.6), Plain white fish (55.6), Biltong (55.6), Droëwors (55.6), Bobotie (22.2)
Carbohydrates	Porridge (oats/maizemeal) (100.0), Cereals (not frosted/high sugar e.g., Weetabix) (100.0), Pap (88.9), Rice (88.9), Samp and beans (77.8), Pasta (plain) (77.8), White bread (77.8), Brown bread (77.8), Chips (hot chips) (66.7), Pizza (66.7), Vetkoek (66.7), Crisps (55.6)
Liquids and dairy	Yoghurt (100.0), Fruit juice (e.g., Liquifruit/Oros) (88.9), Milk (88.9), Hard cheese (e.g., cheddar) (77.8), Amasi (66.7), Processed cheese (e.g., Baby Bells/cheese strings) (66.7), Cream cheese (e.g., Kiri) (66.7), Rooibos tea (66.7)
Vegetables	Cabbage (100.0), Green beans (100.0), Peas (100.0), Sweet potatoes (88.9), Broccoli (88.9), Cooked carrots (88.9), Onions (88.9), Salad greens (66.7), Cucumber (66.7), Mushrooms (66.7), Cooked peppers (66.7), Fresh tomatoes (66.7), Tinned tomatoes (66.7), Cauliflower (66.7), Sweetcorn (55.6), Raw carrots (55.6), Chakalaka (55.6), Raw peppers (44.4), Parsnips (33.3)
Fruit	Bananas (100.0), Oranges (88.9), Peaches/nectarines (88.9), Pears (88.9), Mango (88.9), Other fresh fruit (88.9), Strawberries (88.9), Grapes (77.8), Pineapple (77.8), Apples (raw) (77.8), Apples (baked/pureed) (66.7), Melon (66.7), Kiwi (44.4)
Snacks	Sweets (e.g., lollipops, gummy sweets) (88.9), Cake (77.8), Biscuits (77.8), Ice cream (77.8), Chocolate (77.8)

Analysis of the Food Preference Questionnaire indicated that most children had tried and consumed a wide range of commonly available foods across all food groups. Overall, staple carbohydrates, common protein sources, dairy products and several fruits were consumed by most children, while consumption of some vegetables and traditional dishes was more variable. Across the carbohydrate group, the food consumed by more than 80% of children were porridge (oats/maizemeal) (100%), cereals (not frosted/high sugar e.g., Weetabix) (100%), pap (88.9%) and rice (88.9%). This suggests that children's diets were largely centred around refined carbohydrates and convenience foods, which are often more affordable and accessible in many South African households. Within meats and protein sources, beef (100%), chicken (100%) and eggs (100%) were eaten by all children. However, several other protein sources such as pork and fish were only consumed by 55.6% of children, suggesting variability in protein intake across households. Furthermore, preparation of meat was also noted by caregivers as being hard to swallow and many caregivers reported on modifying texture through cutting or blending.

For liquids and dairy, milk yoghurt (100%) was consumed by all children. High consumption was also reported for fruit juice (88.9%) and milk (88.9%). Consumption of vegetables varied across items. Cabbage (100%), green beans (100%) and peas (100%) were eaten by all children, while sweet potatoes (88.9%), broccoli (88.9%), cooked carrots (88.9%) and onions (88.9%) were also widely consumed. Fruit consumption was generally high. Bananas (100%) were eaten by all children, while oranges (88.9%), peaches or nectarines (88.9%), pears (88.9%), mangoes (88.9%), other fresh fruit (88.9%) and strawberries (88.9%) were also widely consumed. In South Africa, access to fruit is widely available and speaks to participants' access to different options eaten. Finally, snack foods were widely consumed, with sweets (88.9%) reported most frequently. These patterns may reflect the influence of food affordability, household preferences and the feeding adaptations required for children with CP, particularly where softer or energy‐dense foods are easier to prepare and consume.

Based on HFIAS responses, seven households (77.8%) experienced food access constraints, including anxiety about food sufficiency and inability to purchase appropriate foods. Monthly household food expenditure varied widely, with most caregivers reporting spending between $56 and $140 (R1000–R2,500) or $140 and $280 (R2,500–R5,000), and two caregivers reporting expenditures above $280 (R5,000) despite low or absent household income. Reported food expenditure did not consistently align with employment status or receipt of social grants, indicating reliance on informal financial support such as family assistance or community food donations. One caregiver described supplementing grant income with family assistance, explaining, ‘I'm using my UIF … and I have money from the disability grant … and my mother … my mother is helping’ (P5). Seven caregivers (77.8%) reported that disability grant income was insufficient to meet household food needs, particularly when accounting for the child's feeding requirements. One caregiver explained, ‘The grant helps, but it is not enough for food the whole month’ (P2). Five caregivers (55.6%) reported receiving intermittent community food support, primarily in the form of food parcels, which were described as inconsistent. One caregiver stated, ‘We only received the food parcel once, that month it helped’ (P4).

### 3.4 Food Utilization

Food utilization played a critical role, as this directly impacted which foods children were able to consume. Children with greater feeding and functional impairments experienced heightened vulnerability. Those classified at higher EDACS levels (III–V) (*n* = 5, 55.6%) reported greater difficulty eating available foods that were able to meet their nutritional needs. In addition to food type, mealtime duration emerged as an important consideration. Caregivers identified prolonged mealtimes between 30 and 90 min as reported by two caregivers ‘It can take even like 40 minutes feeding her’ while another shared, ‘I have to spend more than an hour and 30 minutes … until he can really eat’ (P8). All caregivers (100%) reported adapting foods through texture modifications and preparation changes. One caregiver explained, ‘He doesn't eat solid food. It's only soft, we have to blend everything for him … Everything we blend’ (P8). Another caregiver described steaming and blending meals to preserve nutritional value while ensuring safe administration, stating, ‘I've got a steamer … I steam the stuff, or I boil it … and then I've got a hand blender. I blend it … We have to mix it actually with formula milk to make it as liquid as possible’ (P7). These accounts illustrate not only the practical implementation of texture modification but also the reliance on specific equipment, ingredients and resources to enable safe feeding.

Food inventory data showed limited dietary diversity in six households (66.7%). Foods that all or most children ate included soft staples such as pap, porridge, mashed vegetables, white bread and yoghurt. Foods that many children did not eat included raw vegetables, whole fruits and certain meats due to chewing or swallowing difficulty. Caregivers described restricting food choices to items their child could safely manage. One caregiver stated, ‘I only give foods that I know he won't choke on’ (P6), while another explained, ‘Some foods I just avoid because it's too difficult for her to eat’ (P1). Therefore, additional dimensions to food security were brought in related not only to the foods that the child would eat but also to how they would eat them.

### 3.5 Food Stability

Five caregivers (55.6%) reported fluctuations in food access across the month, with improved access immediately after grant payments and increased constraints toward month‐end. During periods of instability, caregivers described reducing dietary variety and relying on staple foods, as one caregiver explained, ‘At the end of the month, we eat whatever is left’ (P8). Caregivers also reported that rising food costs, unexpected expenses, illness and employment uncertainty disrupted feeding routines over time, contributing to fluctuating household food security. One caregiver reflected on financial instability, stating, ‘Financially things are so bad …’(P7), while another expressed concern about future income security, explaining, ‘Will you still have your job in the future? … Will it be enough?’ (P2). Similarly, a caregiver described periods of economic strain, noting, ‘Sometimes there's a lack of money to buy food … I'm struggling’ (P3). These accounts illustrate how broader economic vulnerability directly shaped the stability of household food access over time.

## 4 Discussion

This study explored how children with CP experience feeding and nutrition within the South African food environment. The findings suggest that feeding challenges for children with CP extend beyond clinical swallowing impairments and are shaped by structural conditions and knowledge that influence food availability, affordability and preparation within households. Interpreted through the dimensions of food security, availability, access, utilization and stability, the results indicate that children with CP experience distinct vulnerabilities across the food system. A food sovereignty perspective further highlights how inequalities within food systems influence which foods are produced, distributed and economically accessible, shaping children's nutritional opportunities (Moyo and Thow 2020; Pillay 2022; Weiler et al. 2015).

A key contribution of this study is demonstrating that feeding challenges are not experienced uniformly among children with CP. Children with greater functional severity (EDACS III–V) were more vulnerable to constraints across the food system. Rather than reflecting individual‐level feeding limitations alone, these differences illustrate how disability interacts with structural inequalities within food environments (Adams et al. 2024; Goh et al. 2018). In contexts where healthcare, specialized feeding services and disability‐responsive nutrition support are limited, functional severity intensifies existing household vulnerabilities (Zhao et al. 2025). Food insecurity for children with CP therefore emerges from the intersection of disability, poverty and structural inequalities within national food systems (Khan et al. 2025; Moyo and Thow 2020).

### 4.1 Feeding Children With CP Within a South African Food Environment

Although food was widely available within formal retail environments, this did not necessarily translate into foods suitable for children with CP. Conventional definitions of food availability focus on the physical presence of food within markets (Sabaté 2019). However, children with CP often require texture‐modified foods, thickened liquids and nutrient‐dense diets to support safe swallowing and growth (Chichevska‐Jovanova et al. 2025; Sleigh 2005). The findings in the current study showed that children's diets appeared to be dominated by staple carbohydrates, commonly available protein sources, dairy products and fruit, while consumption of some vegetables and certain traditional foods varied across participants. These patterns likely reflect the intersection of feeding abilities, caregiver adaptations and the broader household food environment (Moyo and Thow 2020). These findings suggest that children with CP in this study were exposed to a diverse but nutritionally uneven food environment, where staple foods and easily prepared items were most consumed. Dietary patterns appear to be shaped by a combination of caregiver feeding adaptations, child feeding abilities, household affordability, knowledge, equipment and broader food system factors.

Affordability emerged as a key factor shaping the foods available to children with CP. Food purchasing decisions in South African households are strongly influenced by cost (Chakona and Shackleton 2019; Tuomala and Grant 2022). For children with CP, whose diets may require higher energy and protein intake and additional vitamins and minerals due to some medications, financial constraints may limit access to nutritionally appropriate foods (Hillesund et al. 2007; Kuperminc et al. 2013). Although social grants provide important support, previous research indicates that they may not adequately offset the additional costs associated with disability‐related care (Granlund and Hochfeld 2020). Consequently, households may face persistent challenges in securing foods that meet both nutritional and feeding safety requirements. As a result, we see that economic pressures shape dietary patterns in ways that may inadvertently compromise nutritional adequacy, reinforcing what has been described as the double burden of malnutrition in South Africa (Moyo and Thow 2020).

Food utilization is particularly relevant for children with CP because safe ingestion depends on both physiological and environmental factors. Dysphagia, oral–motor impairment and gastrointestinal complications commonly affect feeding in this population (Sleigh 2005; Taylor et al. 2025). Caregivers in this study described modifying food textures and adapting feeding practices to support safe eating. Similar caregiver adaptations have been reported in other LMICs where access to multidisciplinary feeding services is limited (Adams et al. 2012; Ahmadizadeh et al. 2015; Kisinna et al. 2025; Makhoul et al. 2026). In addition, caregivers reported on prolonged mealtimes as a challenge. Previous research has shown that children with cerebral palsy, including those with milder functional impairment such as EDACS I, often demonstrate a reduced dysphagia limit per sip or bite, meaning the amount of food safely swallowed at one time is significantly smaller (Schepers et al. 2022). As a result, the total amount of food consumed within a typical 30‐min mealtime may be substantially reduced, increasing the importance of ensuring that available foods are nutrient‐dense and of high nutritional value. This highlights that food security is not only about access to food quantity but also access to foods that provide sufficient nutritional benefit within the child's swallowing and feeding capacity. These findings highlight the importance of caregiver knowledge and access to professional feeding support in promoting safe food utilization.

Food stability refers to consistent access to adequate food over time (FAO 2008). Caregivers described how rising food prices, employment instability and caregiving demands affected household food purchasing patterns. These experiences reflect broader structural conditions within South Africa, where economic inequality and unemployment contribute to persistent household food insecurity (Chakona and Shackleton 2019; Odunitan‐Wayas et al. 2018). For children with CP, disruptions in food access may have greater consequences because feeding routines often depend on consistent access to specific foods and preparation methods.

While the food security framework highlights structural barriers to nutrition, a food sovereignty perspective emphasizes issues of equity and control within food systems (Moyo and Thow 2020). Disability is rarely considered within food sovereignty discussions. The findings suggest that children with CP occupy a marginal position within current food environments, where affordability, cultural food practices and feeding safety must be negotiated simultaneously (Battersby and Crush 2014; Chakona and Shackleton 2019). In South Africa, the absence of CP‐specific nutritional guidelines and limited professional training on disability‐related food insecurity further compound these challenges (Jacobson et al. 2024; Quarmby and Pillay 2018). Addressing these gaps will require integrating disability considerations into both nutrition policy and food system planning. Without such reforms, nutritional vulnerability and caregiver burden will persist, undermining both child development and family well‐being.

A strength of this research included the use of a mixed methods research design. Nuanced caregiver narrated experiences could be explored alongside the quantitative data obtained which provided better understanding of the results for the researcher. The data collection tools (e.g., GMFCS, EDACS and HFIAS) used in this study have been used in other research studies and have been validated. Tools were also adapted for the South African context such as the food included in the FPQ. However, limitations are acknowledged. The small sample size and use of purposive sampling limit the generalizability of the findings beyond South African children with cerebral palsy and to other disability groups or contexts. As data were collected at a single point in time, the study does not capture changes in experiences over time, particularly understanding the sensitive nature of food security. Additionally, interviews were conducted in English only, which may have restricted participants' expression. Although survey and qualitative data were triangulated and piloted, variation in caregiver interpretation of survey items may have influenced responses. These limitations should be considered when interpreting the findings, which nonetheless provide useful insights to inform practice and highlight the need for further research with larger and more diverse samples across different countries.

## 5 Conclusion

This study demonstrates that caregivers of children with cerebral palsy face layered and intersecting barriers such as good consistency to providing appropriate and nutritious food within constrained food environments. While food may be present at household level, limitations in availability, access, utilization and stability collectively undermine the realization of the right to food for children with CP. The findings highlight the inadequacy of food security frameworks and policy responses that do not explicitly account for disability. Adopting a food sovereignty perspective also highlights the importance of designing food systems that are inclusive of diverse feeding abilities and nutritional requirements. Without such reforms, children with CP will continue to experience disproportionate vulnerability to food insecurity, with implications for growth, development and long‐term health outcomes.

## Author Contributions

**Tiya Vala:** conceptualization, methodology, formal analysis, investigation, data curation, writing – original draft, writing – review and editing. **Radia Ameen:** conceptualization, investigation, writing – original draft, writing – review and editing, formal analysis, data curation, methodology. **Skye Nandi Adams:** conceptualization, data curation, formal analysis, project administration, resources, supervision, validation, writing – review and editing, writing – original draft.

## Funding

The authors have nothing to report.

## Ethics Statement

This study was approved by the University of the Witwatersrand Human Research Ethics Committee (HREC) (Non‐Medical) (Approval number: STA_2025_39).

## Consent

Written consent was obtained from all involved participants.

## Conflicts of Interest

The authors declare no conflicts of interest.

## Supporting information


**Data S1:** Supporting Information.

## Data Availability

The data that support the findings of this study are available from the corresponding author upon reasonable request.
