# Supplementary material for: The Right to Food for Children With Cerebral Palsy in South Africa: Examining Nutritional Access and Structural Barriers
Source: Child Care Health Dev. 2026 Aug 23;52(5):e70323. doi: 10.1111/cch.70323 (PMC13500202; doi:10.1111/cch.70323)
Supplement: Supplementary file 1 [file CCH-52-e70323-s001.pdf]

# Understanding the needs of children with Cerebral palsy who require specialised diets

Please complete the survey below.

Thank you!

Thank you for taking the time to complete this survey. Please provide as much detail as you can. No personal information will be recorded, and your response is CONFIDENTIAL. If you agree to participate in this survey, please respond to the questions and statements below. If you do not wish to participate, you may close this screen and no information will be recorded. If you agree to participate, we ask that you complete the questions and answer freely, please note you may leave out any questions that you do not wish to answer. It should take you no more than 20 minutes to complete the survey. If you have any complaints about any ethical aspects of the research or feel that you have been harmed in any way by participating in this study, please get in touch with Shaun Schoeman, Shaun.Schoeman@wits.ac.za - 011717 1408
I agree to participate in this survey voluntarily. I acknowledge that should I wish to withdraw from this research at any point I may abandon completing the survey and close this screen with no information recorded. By participating in this study, you acknowledge that you have read and understood the information provided in this document. Your voluntary participation is greatly appreciated.

- ◯ I provide informed consent and agree to participate.
- ◯ I do not provide informed consent and do not agree to participate.

## Demographic Questionnaire

What is your age? ______________________

What is your relationship with the child? ______________________

What is your employment status?
- ◯ Employed full-time
- ◯ Employed part-time
- ◯ Unemployed
- ◯ Unemployed and seeking employment

What is your current job description? ______________________

Estimate total monthly income?
- ◯ Less than  R2000
- ◯ R2000-R5000
- ◯ R5000-R10, 000
- ◯ R10, 000-R20,000
- ◯ More than R20,000

Who lives with the family and what are their ages? ______________________

Do you receive a government grant?
- ◯ Yes
- ◯ No

Please specify : ______________________

| | |
|---|---|
| What is the child's age? | __________________________________ |
| What is the child's gender? | ◯ Female<br>◯ Male |
| Does the child follow a special diet?<br>If yes please specify. | __________________________________________ |
| What's the primary feeding concerns for the child? | ☐ Difficulty Chewing<br>☐ Difficulty Swallowing<br>☐ Risk of aspiration<br>☐ Limited appetite<br>☐ Other |
| Please specify "other" primary concerns: | __________________________________________ |
| What interventions or strategies are currently used for feeding the child? | ☐ Gastronomy Tube Feeding<br>☐ Modified Textures (e.g. Puree or thick liquids)<br>☐ Modified Utensils (e.g. nosey cups - to support safe and easier feeding)<br>☐ Feeding posture adjustment<br>☐ Pacing and cueing (slower feeding with prompts to ensure safe swallows)<br>☐ Environmental control (quiet, focused space - to support mealtime regulation)<br>☐ Other interventions or strategies |
| Please specify "other interventions/ strategies": | __________________________________________ |

## Gross Motor Function Scale (GMFCS)

| | |
|---|---|
| Please choose an age range for your child | ◯ Aged 2 to < 4 Years<br>◯ Aged 4 to < 6 Years<br>◯ Aged 6 to < 12 Years |
| Please select the option that best describes your child's movement:<br>My child has difficulty controlling head and trunk posture in most positions | ◯ and uses specially adapted seating to sit comfortably<br>◯ and has to be lifted by another person to move about<br>◯ Not Applicable |
| Please select the option that best describes your child's movement:<br>My child can sit on own when placed on the floor and can move within a room | ◯ and uses hands for support to maintain sitting balance<br>◯ and usually uses adaptive equipment for sitting and standing<br>◯ and moves by rolling, creeping on stomach or crawling<br>◯ Not Applicable |

| | |
|---|---|
| Please select the option that best describes your child's movement:<br>My child can sit on own and walk short distances with a walking aid (such as a walker, rollator, crutches, canes, etc.) | ◯ and may need help from an adult for steering and turning when walking with an aid<br>◯ and usually sits on floor in a "W-sitting" position<br>◯ and may need help from an adult to get into sitting<br>◯ and may pull to stand and cruise short distances<br>◯ and prefers to move by creeping and crawling<br>◯ Not Applicable |
| Please select the option that best describes your child's movement:<br>My child can sit on own and usually moves by walking with a walking aid | ◯ and may have difficulty with sitting balance when using both hands to play<br>◯ and can get in and out of sitting positions on own<br>◯ and can pull to stand and cruise holding onto furniture<br>◯ and can crawl, but prefers to move by walking<br>◯ Not Applicable |
| Please select the option that best describes your child's movement:<br>My child can sit on own and moves by walking without a walking aid | ◯ and is able to balance in sitting when using both hands to play<br>◯ and can move in and out of sitting<br>◯ and standing positions without help from an adult<br>◯ and prefers to move by walking.<br>◯ Not Applicable |
| Please select the option that best describes your child's movement:<br>My child has difficulty sitting on their own and controlling their head and body posture in most positions | ◯ and has difficulty achieving any voluntary control of movement<br>◯ and needs a specially-adapted supportive chair to sit comfortably<br>◯ and has to be lifted or hoisted by another person to move<br>◯ Not Applicable |
| Please select the option that best describes your child's movement:<br>My child can sit on their own but does not stand or walk without significant support and adult supervision | ◯ and may need extra body/trunk support to improve arm and hand function<br>◯ and usually needs adult assistance to get in and out of a chair<br>◯ and may achieve self mobility using a powered wheelchair or is transported in the community<br>◯ Not Applicable |
| Please select the option that best describes your child's movement:<br>My child can walk on their own using a walking aid (such as a walker, rollator, crutches, canes, etc.) | ◯ and can usually get in and out of a chair without adult assistance<br>◯ and may use a wheelchair when travelling long distances or outside<br>◯ and find it difficult to climb today is a walk on an uneven surface without considerable help<br>◯ Not Applicable |
| Please select the option that best describes your child's movement:<br>My child can walk on their own without using a walking aid, but has difficulty walking long distances or uneven surfaces | ◯ and can sit in a normal adult chair and use both hands freely<br>◯ and can move from the floor to stand in without adult assistance<br>◯ and needs to hold the handrail when going down or upstairs<br>◯ and is not yet able to run and jump<br>◯ Not Applicable |

| | |
|---|---|
| Please select the option that best describes your child's movement:<br>My child can walk on their own without using a walk aid, including fairly long distances, outdoors and on uneven surfaces | ◯ and can move from the floor or a chair to standing without using their hands for support<br>◯ and can go up and down stairs without needing to hold the handrail<br>◯ and is beginning to run and jump<br>◯ Not Applicable |
| Please select the option that best describes your child's movement:<br>My child has difficulty sitting on their own and controlling their head and body posture in most positions | ◯ and has difficulty achieving any voluntary control of movement<br>◯ and needs a specially-adapted supportive chair to sit comfortably<br>◯ and has to be lifted or hoisted by another person to move<br>◯ Not Applicable |
| Please select the option that best describes your child's movement:<br>My child can sit on their own but does not stand or walk without significant support | ◯ and therefore relies mostly on wheelchair at home, school and in the community<br>◯ and often needs extra body/trunk support to improve arm and hand function<br>◯ and may achieve self mobility using a powered wheelchair<br>◯ Not Applicable |
| Please select the option that best describes your child's movement:<br>My child can't stand on their own and only walks using a walking aid (such as a walker, rollator, crutches, canes, etc.) | ◯ and find it difficult to climb stairs, or walk on uneven surfaces<br>◯ and may use a wheelchair when travelling for long distances or in crowds<br>◯ Not Applicable |
| Please select the option that best describes your child's movement:<br>My child and walk on their own without using walking aids, but needs to hold the handrail when going up or down stairs | ◯ and often find a difficult to walk on uneven services, slopes or in crowds<br>◯ Not Applicable |
| Please select the option that best describes your child's movement:<br>My child can walk on their own without using walking aid and can go up or down stairs without needing to hold the handrail | ◯ and walks wherever they want to go (include an uneven surfaces, slopes or in crowds<br>◯ and can run and jump although the speed, balance, and coordination may be slightly limited<br>◯ Not Applicable |

## Eating and Drinking Ability Classification System (EDACS)

| | |
|---|---|
| 1. How would you describe your child 's ability to eat food? | ◯ Eats and drinks safely and efficiently (No difficulty with biting, chewing, or swallowing)<br>◯ Eats and drinks safely but with some limitations in efficiency (May take longer to eat or require some minor modifications, such as softer food)<br>◯ Eats and drinks with some limitations in safety (Occasionally coughs or chokes while eating; needs more significant modifications)<br>◯ Eats and drinks with significant limitations in safety and efficiency (Frequent coughing or choking, requires special food textures or thickened liquids)<br>◯ Unable to eat and drink safely (Needs alternative feeding methods such as a feeding tube) |

| | |
|---|---|
| 2. Does your child require food texture modifications to eat safely? | ◯ No modifications needed<br>◯ Some minor modifications (e.g., softer foods)<br>◯ Moderate modifications (e.g., mashed or finely chopped food)<br>◯ Major modifications (e.g., pureed food only)<br>◯ Unable to eat orally; requires a feeding tube |
| 3. How does your child handle drinking liquids? | ◯ Drinks all types of liquids without difficulty<br>◯ Needs some modifications (e.g., slower drinking, smaller sips)<br>◯ Needs thickened liquids to avoid choking<br>◯ Unable to drink safely; requires tube feeding |
| 4. Does your child cough, gag, or choke while eating or drinking? | ◯ Never<br>◯ Occasionally<br>◯ Frequently<br>◯ Almost |
| 5. How much assistance does your child need during mealtimes? | ◯ Eats independently<br>◯ Requires some supervision or prompting<br>◯ Requires assistance with some parts of eating (e.g., cutting food, holding utensils)<br>◯ Requires full assistance for all meals |
| 6. Does your child experience discomfort or distress while eating or drinking? | ◯ No discomfort<br>◯ Some discomfort occasionally<br>◯ Regular discomfort (e.g., difficulty swallowing, reflux, pain)<br>◯ Severe discomfort; refuses to eat |
| 7. Does your child use any special feeding equipment? (Select all that apply) | ☐ No special equipment needed<br>☐ Special utensils (e.g., adapted spoons, non-slip bowls)<br>☐ Special cups (e.g., spill-proof, straw cups)<br>☐ Feeding tube (e.g., gastrostomy tube) |

## Household Food Insecurity Access Scale (HFIAS)

| | |
|---|---|
| 1. In the past four weeks, did you worry that your household would not have enough food? | ◯ No<br>◯ Yes |
| How often did this happen? | ◯ Rarely (once or twice in the past four weeks)<br>◯ Sometimes (3 to 10 times in the past four weeks)<br>◯ Often (more than 10 times in the past four weeks) |
| 2. In the past four weeks, were you or any household member not able to eat the kind of food you preferred because of a lack of resources? | ◯ No<br>◯ Yes |
| How often did this happen? | ◯ Rarely (once or twice in the past four weeks)<br>◯ Sometimes (3 to 10 times in the past four weeks)<br>◯ Often (more than ten times in the past four weeks) |
| 3. In the past four weeks did you or any household member have to eat a limited variety of food due to a lack of free resources? | ◯ No<br>◯ Yes |

| | |
|---|---|
| How often did this happen? | ◯ Rarely (once or twice in the past four weeks)<br>◯ Sometimes (3 to 10 times in the past four weeks)<br>◯ Often (more than ten times in the past four weeks) |
| 4. In the past four weeks did you or any household member have to eat some foods that you really did not want to eat because of a lack of resources to obtain other types of food? | ◯ No<br>◯ Yes |
| How often did this happen? | ◯ Rarely (once or twice in the past four weeks)<br>◯ Sometimes (3 to 10 times in the past four weeks)<br>◯ Often (more than ten times in the past four weeks) |
| 5. In the past four weeks did you or any household member have to eat a smaller meal than you felt you needed because there was not enough food? | ◯ No<br>◯ Yes |
| How often did this happen? | ◯ Rarely (once or twice in the past four weeks)<br>◯ Sometimes (3 to 10 times in the past four weeks)<br>◯ Often (more than ten times in the past four weeks) |
| 6. In the past weeks, did you or any other household made that have to eat fewer meals in a day because there was not enough food? | ◯ No<br>◯ Yes |
| How often does this happen? | ◯ Rarely (once or twice in the past four weeks)<br>◯ Sometimes (3 to 10 times in the past four weeks)<br>◯ Often (more than ten times in the past four weeks) |
| 7. In the past weeks, was there ever no food to eat of any kind in your household because of a lack of resources to get food? | ◯ No<br>◯ Yes |
| How often did this happen? | ◯ Rarely (once or twice in the past four weeks)<br>◯ Sometimes (3 to 10 times in the past four weeks)<br>◯ Often (more than ten times in the past four weeks) |
| 8. In the past four weeks, did you or any household member go to sleep at night hungry because there was not enough food? | ◯ No<br>◯ Yes |
| How often did this happen? | ◯ Rarely (once or twice in the past four weeks)<br>◯ Sometimes (3 to 10 times in the past four weeks)<br>◯ Often (more than ten times in the past four weeks) |
| 9. In the past four weeks, did you or any household member go a whole day and night without eating anything because there was not enough food? | ◯ No<br>◯ Yes |
| How often did this happen? | ◯ Rarely (once or twice in the past four weeks)<br>◯ Sometimes (3 to 10 times in the past four weeks)<br>◯ Often (more than ten times in the past four weeks) |

**Food Preference Questionnaire**

| | Has never tried | Dislikes a lot | Dislikes | Neither dislikes nor likes | Likes | Likes a lot |
|---|---|---|---|---|---|---|
| Beef | ○ | ○ | ○ | ○ | ○ | ○ |
| Beef burgers | ○ | ○ | ○ | ○ | ○ | ○ |
| Lamb | ○ | ○ | ○ | ○ | ○ | ○ |
| Pork | ○ | ○ | ○ | ○ | ○ | ○ |
| Chicken | ○ | ○ | ○ | ○ | ○ | ○ |
| Chicken nuggets | ○ | ○ | ○ | ○ | ○ | ○ |
| Bacon | ○ | ○ | ○ | ○ | ○ | ○ |
| Ham | ○ | ○ | ○ | ○ | ○ | ○ |
| Sausage | ○ | ○ | ○ | ○ | ○ | ○ |
| Boerewors | ○ | ○ | ○ | ○ | ○ | ○ |
| Bobotie | ○ | ○ | ○ | ○ | ○ | ○ |
| Fried fish in batter/breadcrumbs (e.g fish fingers) | ○ | ○ | ○ | ○ | ○ | ○ |
| Plain white fish (e.g. cod, haddock) | ○ | ○ | ○ | ○ | ○ | ○ |
| Tinned tuna | ○ | ○ | ○ | ○ | ○ | ○ |
| Biltong | ○ | ○ | ○ | ○ | ○ | ○ |
| Droëwors | ○ | ○ | ○ | ○ | ○ | ○ |
| Eggs | ○ | ○ | ○ | ○ | ○ | ○ |
| Chakalaka | ○ | ○ | ○ | ○ | ○ | ○ |
| Broccoli | ○ | ○ | ○ | ○ | ○ | ○ |
| Cabbage | ○ | ○ | ○ | ○ | ○ | ○ |
| Cauliflower | ○ | ○ | ○ | ○ | ○ | ○ |
| Green beans | ○ | ○ | ○ | ○ | ○ | ○ |
| Mushrooms | ○ | ○ | ○ | ○ | ○ | ○ |
| Onions | ○ | ○ | ○ | ○ | ○ | ○ |
| Parsnips | ○ | ○ | ○ | ○ | ○ | ○ |
| Peas | ○ | ○ | ○ | ○ | ○ | ○ |
| Salad greens (e.g., lettuce) | ○ | ○ | ○ | ○ | ○ | ○ |
| Sweet potatoes | ○ | ○ | ○ | ○ | ○ | ○ |
| Sweetcorn | ○ | ○ | ○ | ○ | ○ | ○ |
| Cucumber | ○ | ○ | ○ | ○ | ○ | ○ |
| Raw carrots | ○ | ○ | ○ | ○ | ○ | ○ |
| Cooked carrots | ○ | ○ | ○ | ○ | ○ | ○ |
| Raw peppers (red/green) | ○ | ○ | ○ | ○ | ○ | ○ |
| Cooked peppers (red/green) | ○ | ○ | ○ | ○ | ○ | ○ |
| Fresh tomatoes | ○ | ○ | ○ | ○ | ○ | ○ |

| | | | | | | |
|---|---|---|---|---|---|---|
| Tinned tomatoes (e.g., as pasta sauce) | ○ | ○ | ○ | ○ | ○ | ○ |
| Apples (raw) | ○ | ○ | ○ | ○ | ○ | ○ |
| Apples (baked or pureed) | ○ | ○ | ○ | ○ | ○ | ○ |
| Bananas | ○ | ○ | ○ | ○ | ○ | ○ |
| Oranges | ○ | ○ | ○ | ○ | ○ | ○ |
| Grapes | ○ | ○ | ○ | ○ | ○ | ○ |
| Melon | ○ | ○ | ○ | ○ | ○ | ○ |
| Peaches, nectarines | ○ | ○ | ○ | ○ | ○ | ○ |
| Pears | ○ | ○ | ○ | ○ | ○ | ○ |
| Strawberries | ○ | ○ | ○ | ○ | ○ | ○ |
| Mango | ○ | ○ | ○ | ○ | ○ | ○ |
| Kiwi fruit | ○ | ○ | ○ | ○ | ○ | ○ |
| Pineapple | ○ | ○ | ○ | ○ | ○ | ○ |
| Other fresh fruit | ○ | ○ | ○ | ○ | ○ | ○ |
| Cake | ○ | ○ | ○ | ○ | ○ | ○ |
| Biscuits | ○ | ○ | ○ | ○ | ○ | ○ |
| Ice cream | ○ | ○ | ○ | ○ | ○ | ○ |
| Chocolate | ○ | ○ | ○ | ○ | ○ | ○ |
| Sweets (e.g., lollipops, gummy sweets) | ○ | ○ | ○ | ○ | ○ | ○ |
| White bread | ○ | ○ | ○ | ○ | ○ | ○ |
| Brown bread | ○ | ○ | ○ | ○ | ○ | ○ |
| Vetkoek | ○ | ○ | ○ | ○ | ○ | ○ |
| Pap | ○ | ○ | ○ | ○ | ○ | ○ |
| Samp and beans | ○ | ○ | ○ | ○ | ○ | ○ |
| Porridge (Oats/Maizemeal) | ○ | ○ | ○ | ○ | ○ | ○ |
| Cereals (not frosted or high sugar e.g. Weetabix) | ○ | ○ | ○ | ○ | ○ | ○ |
| Chips (hot chips) | ○ | ○ | ○ | ○ | ○ | ○ |
| Rice | ○ | ○ | ○ | ○ | ○ | ○ |
| Pasta (plain) | ○ | ○ | ○ | ○ | ○ | ○ |
| Pizza | ○ | ○ | ○ | ○ | ○ | ○ |
| Crisps | ○ | ○ | ○ | ○ | ○ | ○ |
| Rooibos Tea | ○ | ○ | ○ | ○ | ○ | ○ |
| Fruit Juice (e.g. Liquifruit/ Oros) | ○ | ○ | ○ | ○ | ○ | ○ |
| Milk | ○ | ○ | ○ | ○ | ○ | ○ |
| Yogurt | ○ | ○ | ○ | ○ | ○ | ○ |
| Amasi | ○ | ○ | ○ | ○ | ○ | ○ |
| Cheese (processed e.g. Baby Bells, cheese strings) | ○ | ○ | ○ | ○ | ○ | ○ |

| | | | | | | |
|---|---|---|---|---|---|---|
| Cheese ( hard e.g. cheddar) | ◯ | ◯ | ◯ | ◯ | ◯ | ◯ |
| Cheese (cream, e.g Kiri) | ◯ | ◯ | ◯ | ◯ | ◯ | ◯ |

## Food Texture Modifications & Feeding Practices

1. How do you modify your child 's food texture to make it easier to eat? (Select all that apply)
- ☐ Pureeing or blending food
- ☐ Chopping food into smaller pieces
- ☐ Thickening liquids
- ☐ Adding extra moisture to food
- ☐ Softening food by cooking longer
- ☐ Other (please specify):

Other specified modifications: __________________________________

2. What types of equipment do you use to modify your child 's food? (Select all that apply)
- ☐ Blender or food processor
- ☐ Manual masher or sieve
- ☐ Special feeding spoons or adapted utensils
- ☐ Other (please specify):

Other (please specify equipment) __________________________________

3. How often do you need to modify your child 's food texture?
- ◯ Every meal
- ◯ Once a day
- ◯ A few times a week
- ◯ Rarely

4. What are the biggest challenges you face when modifying your child 's food? (Select all that apply)
- ☐ Cost of food preparation tools (blenders, processors, etc.)
- ☐ Cost of commercial thickeners or specialised ingredients
- ☐ Time required for food preparation
- ☐ Difficulty in finding appropriate foods
- ☐ Access to electricity for blending/cooking
- ☐ Other (please specify):

Other (please specify challenges): __________________________________

## Food Purchasing and Spending Patterns

1. On average, how much do you spend on food each month?
- ◯ Less than R1,000
- ◯ R1, 000 - R2,500
- ◯ R2, 500 - R5,000
- ◯ More than R5,000

2. Where do you buy most of your food? (Tick all that apply)
- ☐ Supermarket (e.g., Pick n Pay, Checkers, Spar)
- ☐ Spaza shop/local convenience store
- ☐ Street vendors
- ☐ Butcher
- ☐ Fresh produce market
- ☐ Other (please specify):

Where (please specify):

__________________________________

## Fast Food Consumption

1. How often does your child eat fast food or takeaways (e.g., KFC, McDonald 's, Kota, vetkoek, fried fish, etc.)?

- ◯ Never
- ◯ Once a month
- ◯ 2-3 times per month
- ◯ Once a week
- ◯ More than once a week

2. What types of fast food does your child eat the most? (Tick all that apply)

- ☐ Fried chicken (e.g., KFC, Chicken Licken)
- ☐ Burgers (e.g., McDonald 's, Steers)
- ☐ Kota / Bunny chow
- ☐ Vetkoek
- ☐ Fried fish and chips
- ☐ Pizza
- ☐ Other (please specify):

Please specify the other types of fast food:

__________________________________

3. Where do you usually buy fast food for your child? (Tick all that apply)

- ☐ Takeaway restaurant (e.g., KFC, McDonald 's)
- ☐ Local street vendor
- ☐ Spaza shop
- ☐ Other (please specify):

Where are other places you usually buy fast food?

__________________________________
